# Historia Febris Hecticæ omnis ævi Observata Medica Continens

**Published:** 1785

**Authors:** 


III. Wenceflai Trnka de Krzowitz, S. R. I. Equitis, Medicin. Doct. in Reg. Univerf. Budenfi Pathologiæ Prof. P. O. *Hiftoria Febris Hecticæ omnis ævi obfervata Medica Continens.* 8vo. Vindobonæ, 1783. p. 415.

THE writer of this volume has, with great induftry, brought together a variety of obfervations on the fubject of hectic fever from the works of many ancient and modern authors.

Suppofing, with the generality of fyftematic writers, that hectic fever may exift as an idiopathic difeafe, he divides it into the two fpecies of *Protopathic* or Primary, and *Deuteropathic* or Secondary. This diftinction, however, we are of opinion, has no foundation in nature, as we think, with Dr. Cullen, that hectic fever is conftantly an effect of fome topical affection,

 moft moſt commonly of an internal ſuppuration. But notwithſtanding this objection, and ſome others which might perhaps be reaſonably offered to points of theory in the courſe of the volume, the work is certainly a uſeful one.

It is divided into two parts; in the firſt, the author treats of the nature and variety of hectic fever; and in the ſecond, of the method of treating it. In this latter part, we find him confirming the efficacy of agaric in diminiſhing the colliquative ſweats of phthiſical patients. He firſt adminiſtered this remedy on the authority of de Haen (*Rat. Med.* p. 12. c. 6. §. 6.) in the doſe of ten grains at bed time, and he has ſince repeatedly experienced its good effects in checking the diſpoſition to ſweat, without occaſioning any increaſe of evacuation by ſtool.

